# Suture button systems for coronoid fracture fixation: a biomechanical time-zero pilot study

**DOI:** 10.1186/s12891-024-08209-z

**Published:** 2025-01-09

**Authors:** Sebastian Lappen, Pavel Kadantsev, Daniel Bohnet, Stephanie Geyer, Maximilian Hinz, Christian Marx, Sepp Braun, Sebastian Siebenlist

**Affiliations:** 1 Department of Sports Orthopaedics, Klinikum rechts der Isar (Technical University of Munich), Ismaninger Straße 22, 81675 Munich, Germany; 2 Department for Orthopedics, St. Vinzenz Klinik, Pfronten, Germany; 3 Research Unit for Orthopaedic Sports Medicine and Injury Prevention, Medical Informatics and Technology, University for Health Sciences, Hall, Austria; 4 Gelenkpunkt, Sports and Joint Surgery Innsbruck, Innsbruck, Austria

**Keywords:** Proximal ulna, Fracture management, Osteosynthesis, Biomechanics, Suture fixation, Elbow

## Abstract

**Purpose:**

This study aims to describe a fixation technique for coronoid fractures using suture buttons, and to biomechanically evaluate this technique in comparison to screw fixation as a time-zero pilot study.

**Methods:**

An O’Driscoll type 2 anteromedial coronoid facet (AMCF) fracture was simulated in 20 fresh-frozen human elbows. The specimens were randomized into two groups and fracture fixation was performed with either a suture button system or a 3.5 mm cannulated screw. Ultimate load-to-failure (N) was then tested for each specimen.

**Results:**

The mean load-to-failure was 322.6 ± 75.9 N for suture button fixation and 314.2 ± 85.9 N for screw fixation. The differences were not statistically significant (*p* = 0.432). Additional fracturing of the coronoid fragment was observed in two specimens with screw fixation.

**Conclusion:**

Promising biomechanical evaluations show that this fixation technique using suture buttons in the treatment of coronoid fractures provides equal construct stability as screw fixation. Further studies are required to fully validate this procedure.

## Introduction

When not treated appropriately, coronoid fractures pose a risk of rapid development of post-traumatic osteoarthritis due to chronic elbow instability [1]. However, there is still no consensus on the optimal treatment strategy when surgery is indicated. Plate fixation has been reported to provide good biomechanical stability [2] but requires an open surgical approach. Screw fixation can be used in both open and arthroscopic approaches [3]. However, the disadvantages of screw fixation include the need for intraoperative X-ray controls and subsequent radiation exposure to secure correct fragment reduction.

Suture button fixation techniques are well established in the management of various joint pathologies, such as acromioclavicular joint dislocations [4] or injuries of the syndesmoses [5], as well as in the management of fractures such as avulsion fractures of the posterior cruciate ligament [6], distal clavicular fractures [7], and glenoid fractures [8]. Suture button systems offer several advantages: besides their technical ease of use, they allow for bone sparing fixation, which is particularly beneficial in the fixation of small fracture fragments. With the help of aiming devices and repositioning pliers, the positioning of fragments is simplified, and fewer intraoperative X-ray controls are necessary. Further, promising biomechanical results have been reported for suture button systems in fracture management [9]. However, the treatment of coronoid fractures with a suture button system has not yet been described.

The purpose of this study was therefore to determine the effectiveness and feasibility of suture button fixation for coronoid fractures in a time-zero pilot biomechanical study. It was hypothesized that no significant differences in ultimate load-to-failure would be found compared to standardized screw fixation.

## Materials and methods

**Fig. 1:**
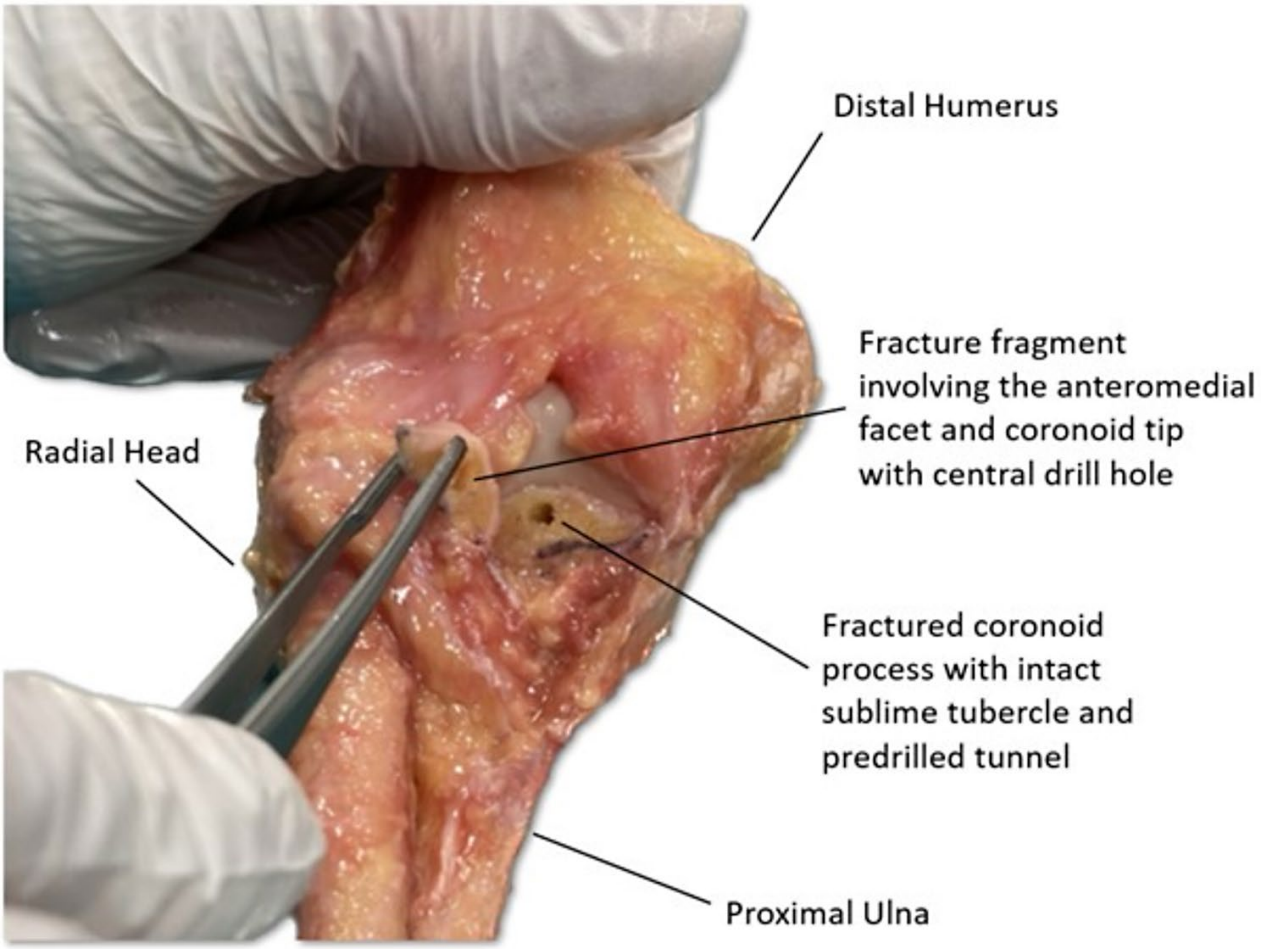
Osteotomy of the coronoid. When performing the osteotomy, care was taken to include the anteromedial facet and tip while leaving the sublime tubercle intact. For suture button or screw fixation, a hole was drilled through the center of the coronoid fragment and a corresponding tunnel was drilled from center of the coronoid osteotomy to the posterior ulna perpendicular to the ulnar shaft

Institutional review board approval was obtained by the ethics committee of the Technical University of Munich prior to commencement of this study (2022-430-S-NP) and the study was performed in accordance with the Declaration of Helsinki. Twenty fresh-frozen cadaver elbows with no evidence of fractures, ligament tears or bony deformities were available for testing. The specimens were thawed for 24 h preceding dissection and testing. Specimens were dissected free of skin and subcutaneous tissue, and all soft tissue was removed except for passive stabilizing structures such as the joint capsule and both collateral ligament complexes. The joint capsule was incised exposing the coronoid, while care was taken not to damage the remaining capsuloligamentous structures. The coronoids were measured using calipers. Osteotomes were then used to simulate anteromedial coronoid facet (AMCF) fractures involving the coronoid tip with intact sublime tubercle (subtype 2 according to O’Driscoll [10]): a transverse osteotomy was set at the center of the coronoid, determined by a line from the tip of the olecranon to the base of the coronoid and running parallel to the longitudinal axis of the ulna (Fig. 1).

The specimens were then randomized to either screw or suture button fixation.

### Screw fixation

**Fig. 2:**
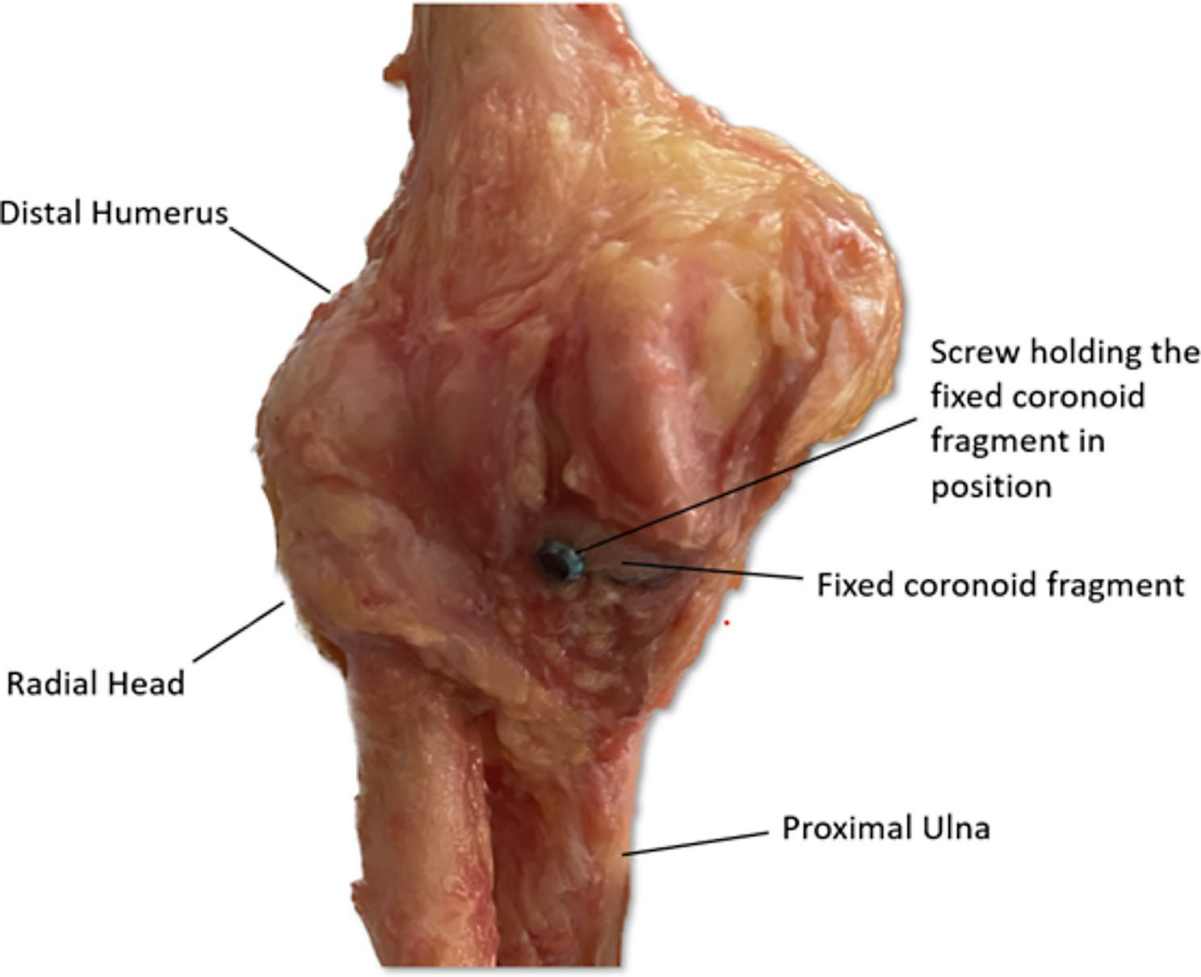
Anatomical positioning of the coronoid fragment with a screw. The screw was inserted bicortically across the fracture

Anterior-to-posterior (AP) screw fixation was performed in screw fixation group as this was found to be biomechanically superior to posterior-to-anterior (PA) screw fixation in a previous study [11]. A 2.0 mm drill tunnel was created perpendicular to the ulnar shaft. The tunnel was drilled through the center of the coronoid fragment and through the center of the coronoid osteotomy, exiting at the posterior ulnar surface. A 3.5-mm cannulated fully threaded screw (DePuy Synthes, Raynham, USA) was then inserted bicortically in AP direction across the fracture (Fig. 2). A screw-to-bone ratio of at least 1:2 was chosen [12].

### Suture button fixation

**Fig. 3:**
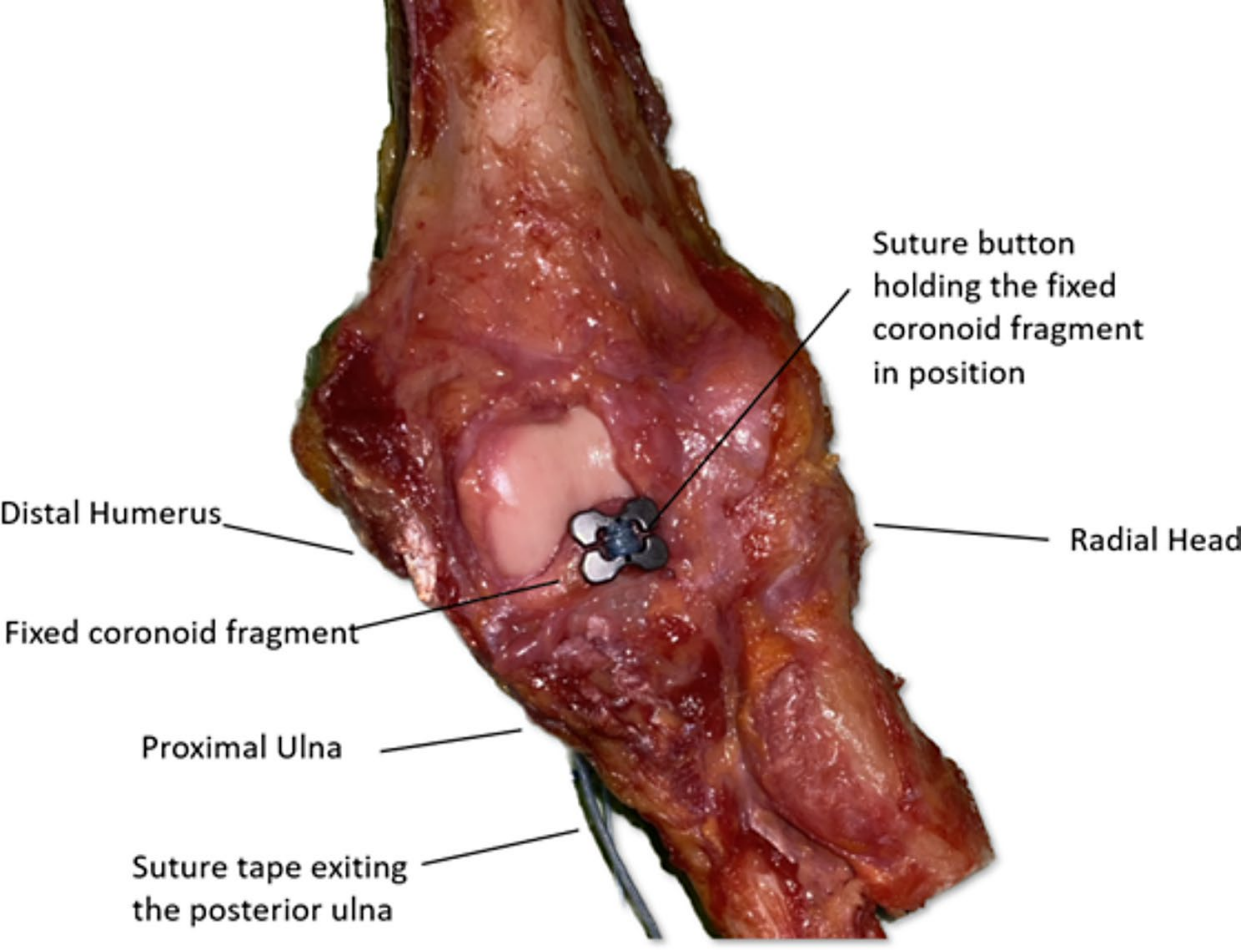
Anatomical repositioning of the coronoid fragment with the Dog-Bone® system. One button was loaded with suture tape which was passed through the predrilled tunnel and then tied over a second suture button on the posterior surface of the ulna

A 2.4 mm bone tunnel was created in the same manner as described above. A cortical button (Dog-Bone®; Arthrex Inc. Naples. USA) was preloaded with two strands of 2 mm suture tape (FiberTape®; Arthrex Inc. Naples. USA). The ends of the suture tape were first passed through the predrilled hole in the coronoid fragment and the button was pulled onto the surface of the coronoid fragment obtaining maximal bone-button contact. The ends of the sutures were then passed through the bone tunnel from anterior to the posterior surface of the ulna. The coronoid fragment was pulled onto the osteotomy. The tails of the two suture tapes were then threaded through a second cortical button, which was placed onto the posterior surface of the ulna, and the suture tapes were then tied over the posterior button with six half-strokes knots. (Fig. 3).

### Biomechanical testing

**Fig. 4:**
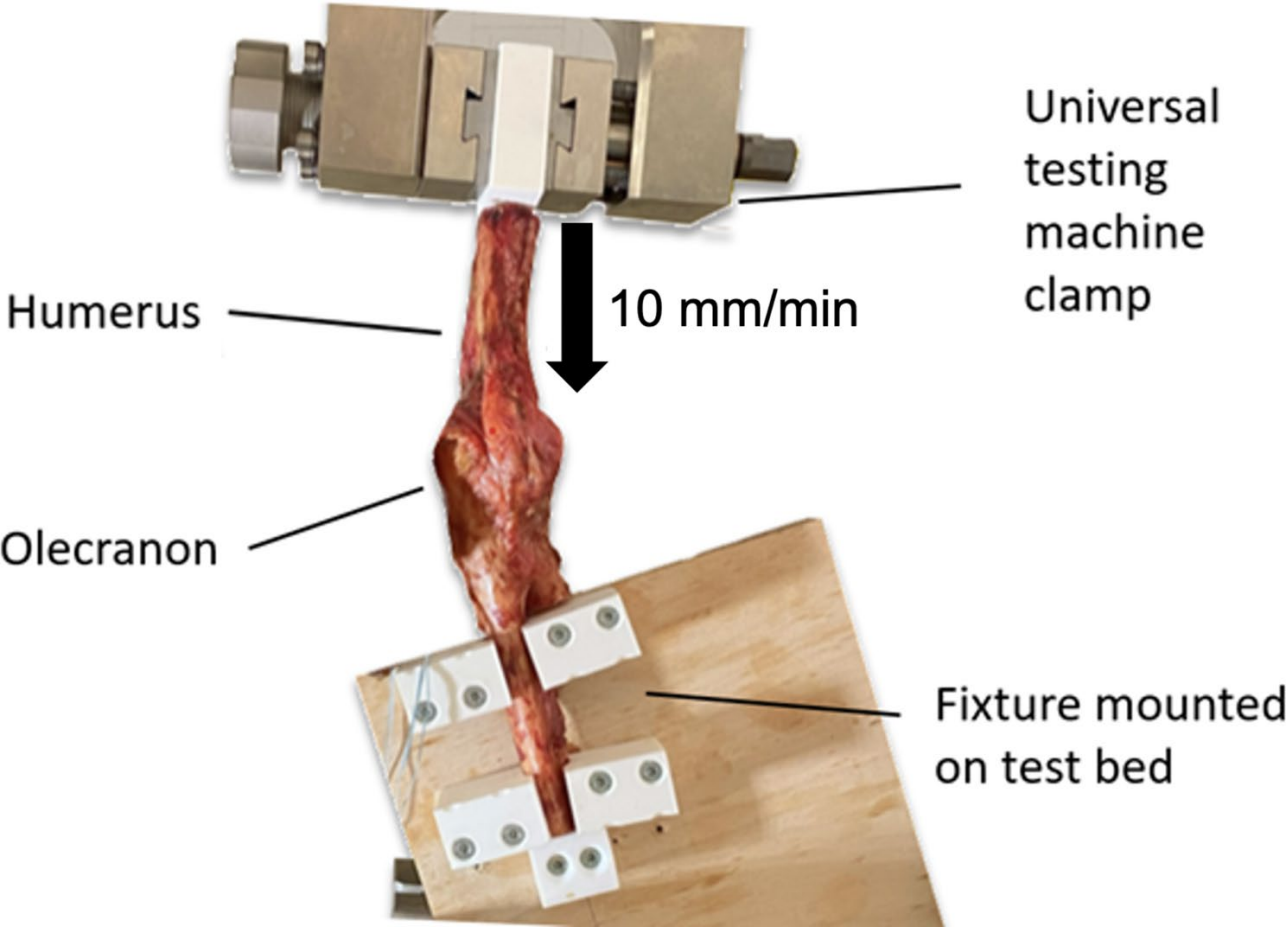
Positioning of the specimens in the custom-made testing setup. This setting mimics a fall on the outstretched hand in which axial force is applied to the elbow. A progressively loaded at a rate of 10 mm/min was applied

The specimens were fixed to a custom jig and secured to the actuator of the material testing machine (Z010 Test Controll II, ZwickRoell, Ulm, Germany). Care was taken to apply posteromedial rotational stress during positioning of the specimen. This setup was chosen because the typically reported mechanism of injury of AMCF fractures is a fall on the outstretched hand with posteromedial rotational force applied to the elbow [10, 13, 14] (Fig. 4).

The coronoid was progressively loaded at a rate of 10 mm/min [15, 16] until a displacement of more than 2 mm [15, 16] occurred at the osteotomy site and the load-to-failure (N) was recorded for each construct.

### Statistical analysis

The sample size was calculated using the G*Power software (latest version 3.1.9.7; Heinrich Heine Universität Düsseldorf, Düsseldorf, Germany). Effect size in terms of maximum failure load, referring to data previously published by Iannuzzi et al. [15], was used to determine differences between two independent means. α was set at o.05. A total samples size of twenty specimens divided into ten specimens per group was required to achieve power of 0.95. All statistical calculations were performed using SPSS Statistics (Version 28, Property IBM Corp., NY, USA). Mann–Whitney U test was used to assess differences between the groups. A value of *p* < 0.05 was considered significant.

## Results

In each group, four male and six female cadavers were used for testing. The suture button group consisted of seven left and three right elbows, while the screw fixation group consisted of one left and nine right elbow joints. The specimens of the groups were comparable in age (suture button fixation: 74.2 years, range, 48–88 years; screw fixation: 77.0 years, range, 48–89 years).

**Fig. 5:**
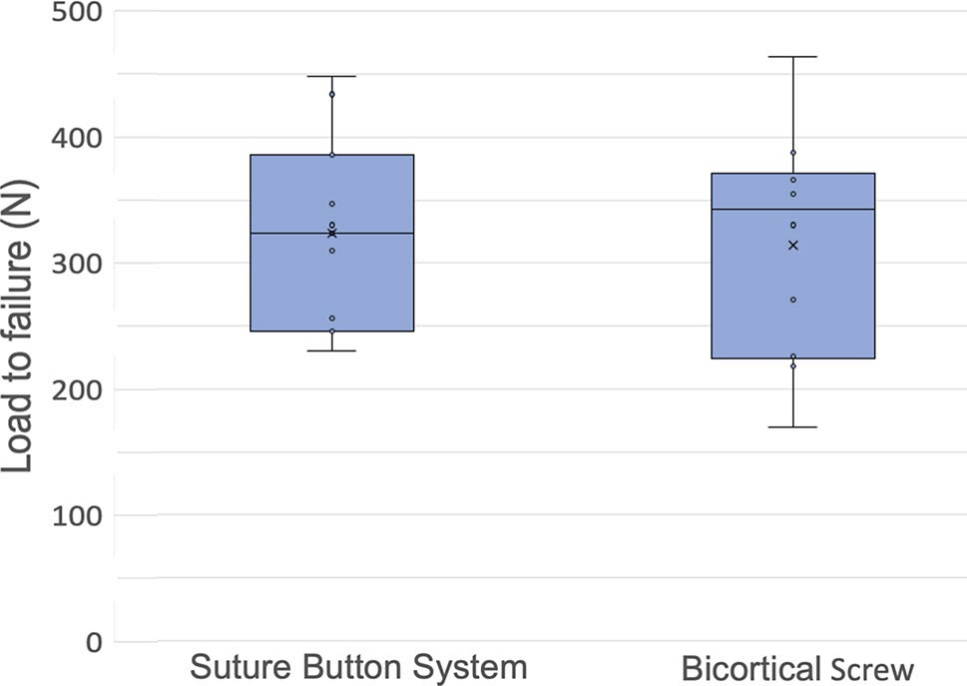
Box plots demonstrating the maximum load to failure

The average failure load was 322.6 ± 75.9 N for suture button fixation and 314.2 ± 85.9 N for screw fixation (Fig. 5).

**Fig. 6:**
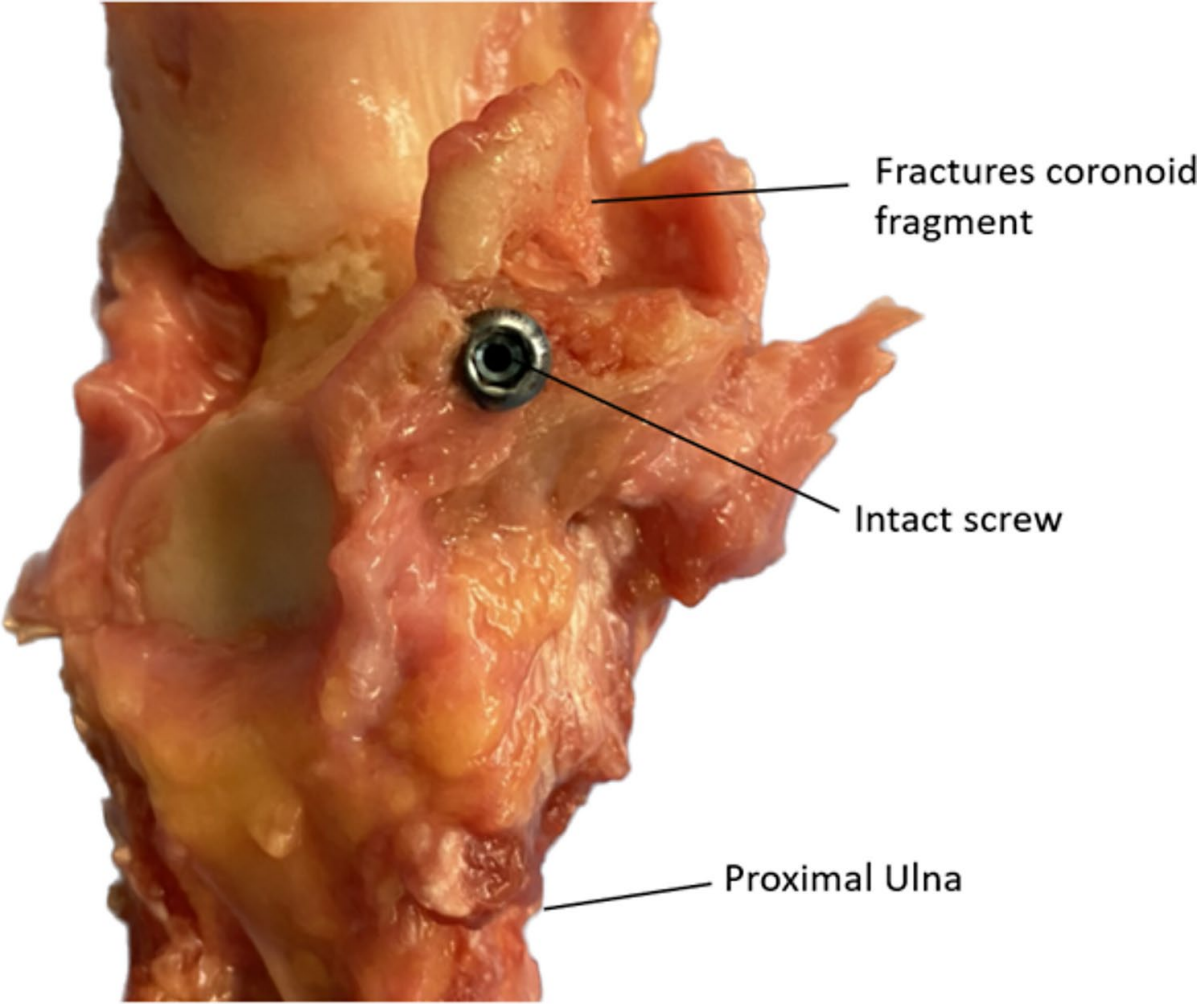
In two cases, a fracture of the coronoid fragment occurred in the screw fixation group

The differences were not statistically significant (*p* = 0.432). All constructs failed due to displacement of the coronoid fragment around an intact fixation system. Suture constructs stretched but did not rupture in all cases, whereas the screws either bent or the coronoid fragment was further fractured in two cases (Fig. 6).

## Discussion

This is the first study to describe and biomechanically evaluate a suture button fixation technique for coronoid fractures. The tests performed showed that the ultimate failure loads after suture button or screw fixation of O’Driscoll subtype 2 fractures of the anteromedial coronoid facet (AMCF) did not differ significantly. This surgical technique therefore presents a promising novel method for the treatment of coronoid fractures.

The present study is the first to describe fixation of coronoid fractures with suture buttons. Failure loads of 314.2 N and 322.6 N were observed after screw fixation and suture button fixation, respectively. These values are comparable to failure loads of coronoid screw fixation reported in previous biomechanical studies [2, 15]. Since dislocation of the fracture fragment after a coronoid fixation is a comparatively rare complication, this fixation force in combination with adequate postoperative rehabilitation appears to be sufficient to avoid failure. However, it must be noted that the present pilot study only measured the static load-to-failure determining the maximum amount of force that the coronoid would experience before construct failure. This model is most comparable to a traumatic event. The displacement under cyclic loading, which reflects the loading of everyday movements in rehabilitation, would be a further important aspect that needs to be evaluated in future studies. Nonetheless, as the biomechanical testing showed equivalent failure loads of the two fixation methods, the suture button fixation technique described appears to be a promising surgical approach. This is consistent with other studies reporting good biomechanical properties of suture button fixation [9, 17–20]. However, the present study should be viewed as the first investigation of a surgical technique that is still being developed and needs to be further investigated.

Furthermore, the presented fixation technique has so far only been tested in subtype 2 AMCF fractures. It therefore still needs to be investigated whether this technique would also show satisfactory results for other coronoid fracture types. It is possible that suture button fixation may show inferior biomechanical properties compared to the screw or plate fixation for larger coronoid fractures. Further, in smaller coronoid fractures, the fracture fragment might crumble during drilling, and the button positioning onto the coronoid fragment might be challenging. Therefore, further investigations of the feasibility and fixation strength of this fixation technique for other coronoid fracture types are necessary.

The fixation of subtype 2 AMCF fractures with suture buttons offers the opportunity to be transferred into an arthroscopic fixation technique. Arthroscopic treatment of coronoid fractures was first described in the year 2007 by Adams et al. [21]. Arthroscopy offers a minimally invasive alternative reducing the risk of complications [3], the rate of which is reported to be up to 25% in the surgical treatment of AMCF fractures [22]. In addition, intra-articular visualization is improved [23] and concomitant intra-articular lesions can directly be addressed [24]. Screw fixation is considered the gold standard for arthroscopic AMCF fracture treatment [25]. Several studies have reported excellent clinical results after arthroscopic screw fixation of O’Driscoll subtype 1 and 2 AMCF fractures [21, 26–31]. However, fracture reduction in particular remains challenging as arthroscopy only allows for indirect screw positioning. The authors therefore believe that there is an unmet need for a novel arthroscopic fixation technique that facilitates fracture reduction. While suture button systems can be more costly than screw fixation, suture button fixation systems offer several advantages over screw fixation. With the help of aiming devices and repositioning pliers, fragment positioning is simplified allowing for fewer intraoperative X-ray checks. Further, they enable a bone-preserving fixation and can be applied in a arthroscopic technique.

Several limitations of this study must be stated. This includes the inherent limitations of in vitro studies as well as the small sample size. However, the number of specimens used is comparable to that of other biomechanical studies on coronoid fixation [15, 16]. In addition, no matched pairs of elbows were available for testing, which would have led to less confounding effects. Although the groups were comparable in age and sex, other characteristics such as bone quality may have varied, and no bone mineral density measurements were performed prior to testing. While the amount of suture tension was controlled by having the same surgeon tie the knot in each trial in the suture button group, tension may differ between surgeons and knot tying techniques in a clinical setting.

## Conclusion

Suture button fixation of coronoid fractures results in equivalent failure loads as screw fixation for subtype 2 AMCF fractures in this time-zero pilot study. This promising technique might help reducing intraoperative X-ray radiation and has the potential to be transferred into an arthroscopic approach. However, future research efforts focusing on further biomechanical and clinical data are needed.

## Data Availability

The datasets used and analysed during the current study are available from the corresponding author on reasonable request.

## Abbreviations

AMCF: Anteromedialcoronoidfacet
AP: Anterior-to-posterior
e.g.: exempli gratio
et al.: et alii / et aliae
N: Newton
PA: Posterior-to-anterior
